# Phenolic-Rich Electrospun Bioactive Nanofibers from Mallow (*Malva sylvestris* L.) as Active Packaging Materials

**DOI:** 10.3390/molecules31183283

**Published:** 2026-09-16

**Authors:** Ayse Saygun

**Affiliations:** Department of Food Engineering, Faculty of Chemical and Metallurgical Engineering, Istanbul Technical University, Maslak, Istanbul 34469, Türkiye; gocmenay@itu.edu.tr; Tel.: +90-5335684414

**Keywords:** *Malva sylvestris* L. (mallow), electrospinning, nanofiber membranes, blueberry, food packaging

## Abstract

The development of bioactive packaging materials represents a promising strategy for reducing postharvest quality losses in fresh produce. In this study, phenolic-rich poly(vinyl alcohol) (PVA) electrospun nanofiber mats incorporating *Malva sylvestris* L. (mallow) leaf extract at 5%, 10%, and 15% were developed and evaluated as active packaging materials for blueberry preservation. The resulting nanofibers were characterized in terms of morphology, phenolic content, antioxidant activity, and antibacterial properties, followed by evaluation of their preservation performance during refrigerated blueberry storage. Scanning electron microscopy revealed smooth, bead-free nanofibers, with mean fiber diameter decreasing from 702 ± 76 nm for the PVA control to 353 ± 38 nm for nanofibers containing 15% extract. Total phenolic content increased significantly with extract loading, reaching 17.76 ± 0.29 mg GAE/g in the 15% formulation. A corresponding concentration-dependent enhancement in antioxidant activity was observed, with the 15% extract-loaded nanofibers exhibiting DPPH and ABTS radical-scavenging activities of 82.9 ± 2.0% and 89.7 ± 2.3%, respectively. The same formulation showed the strongest antibacterial activity among the nanofiber mats, with minimum inhibitory concentrations of 8 μg/mL against *Staphylococcus aureus* and 16 μg/mL against *Escherichia coli*. When applied as an active packaging mat for blueberries stored under refrigerated conditions, the nanofiber treatment markedly delayed visible quality deterioration. After 14 days, nanofiber-packaged blueberries retained a visual-quality score of 4.2 ± 0.45, whereas the untreated control decreased to 1.6 ± 0.55 and was considered commercially unacceptable. Overall, the results demonstrate that incorporation of *M. sylvestris* L. extract into electrospun PVA nanofiber mats provides concentration-dependent phenolic, antioxidant, and antibacterial functionality and contributes to improved maintenance of blueberry visual quality during refrigerated storage. These findings support the potential of *M. sylvestris*-loaded electrospun nanofiber mats as active packaging materials for fresh-produce preservation.

## 1. Introduction

The increasing demand for sustainable food systems has accelerated the development of packaging materials that can reduce dependence on conventional petroleum-based plastics while maintaining food quality and safety. Although plastic packaging provides effective protection against physical damage, moisture transfer, oxidation, and microbial contamination, its persistence in the environment has raised substantial concerns regarding waste accumulation and ecological impact. Consequently, renewable and biodegradable materials with additional preservation functionality have received increasing attention as potential alternatives to conventional food packaging [1,2,3,4].

Active packaging is particularly attractive because it can provide functions beyond passive containment by incorporating compounds capable of interacting with the food or its surrounding environment. Plant-derived antioxidants and antimicrobial compounds are of interest in this context because they can provide multifunctional protection against oxidative and microbial deterioration. Such systems are particularly relevant for fresh fruits, in which postharvest respiration, moisture loss, oxidative reactions, and microbial growth can rapidly reduce quality and marketability [2,3,4,5].

Electrospinning offers a promising platform for incorporating bioactive compounds into polymeric packaging structures. The process generates micro- to nanoscale fibers with a high specific surface area and interconnected porous architecture, providing an efficient matrix for incorporating functional compounds [3,4,5,6,7]. Compared with conventional solvent-cast structures, electrospun materials can provide a larger interfacial area and facilitate interactions between the incorporated compounds and the food environment. These characteristics make electrospinning particularly attractive for the development of active packaging systems containing natural extracts [3,4,5,6]. However, the preservation performance of such systems depends on the compatibility between the polymer matrix, bioactive compounds, and target food, and therefore requires experimental validation rather than being inferred solely from fiber morphology or antioxidant activity.

Polyvinyl alcohol (PVA) is frequently investigated as an electrospinning matrix because of its film-forming ability, aqueous processability, and favorable electrospinnability. PVA can also accommodate a range of plant-derived compounds within electrospun fiber networks, providing a practical platform for developing active packaging materials [4,5,6,7]. Nevertheless, the suitability of PVA-based structures for direct food applications requires careful consideration of their food-contact safety and behavior under actual storage conditions. Therefore, in the present study, the developed PVA structures are considered bioactive/active packaging nanofibers, rather than being designated as edible materials.

Among natural functional ingredients, phenolic-rich plant extracts are particularly promising because of their antioxidant and antimicrobial activities. Phenolic compounds can scavenge reactive species and contribute to oxidative stability, while their interactions with microbial cell membranes and cellular components can inhibit microbial growth [2,5,8]. Incorporating phenolic-rich extracts into electrospun fibers therefore provides an opportunity to combine the structural advantages of a nanofibrous matrix with the biological functionality of plant-derived compounds.

*M. sylvestris* L. (mallow) is a member of the Malvaceae family and represents a relatively underexplored botanical source for active food packaging applications. Its leaves and other plant tissues contain phenolic acids, flavonoids, anthocyanins, tannins, mucilage polysaccharides, and other secondary metabolites [9,10,11]. Previous phytochemical studies have identified compounds such as gallic, caffeic, chlorogenic, ferulic, and *p*-coumaric acids, together with flavonoid derivatives including quercetin and rutin. These constituents have been associated with the antioxidant activity of *M. sylvestris* L. extracts, as demonstrated using commonly applied radical-scavenging and reducing-power assays [9,10]. Extracts of the plant have also demonstrated antimicrobial activity against different microorganisms [10,11]. These characteristics suggest that *M. sylvestris* could serve as a source of multifunctional bioactive compounds for active packaging applications.

The potential value of *M. sylvestris* L. is particularly relevant for highly perishable fruits such as blueberries (*Vaccinium corymbosum* L.). Blueberries are valued for their anthocyanins, flavonoids, phenolic compounds, vitamins, and dietary fiber and are increasingly consumed because of their recognized nutritional and functional properties [12,13,14]. However, their delicate tissues and high moisture content make them susceptible to postharvest deterioration. Respiration, water loss, tissue softening, surface damage, and microbial contamination can progressively reduce visual quality and marketability during storage. In particular, fungal microorganisms, including *Botrytis*, *Alternaria*, *Penicillium*, and *Rhizopus* spp., are important contributors to postharvest deterioration of blueberries [13,14,15]. Refrigerated storage can delay these processes but does not completely prevent quality deterioration.

Active coatings and packaging systems containing natural bioactive compounds have consequently been investigated to improve the storage stability of blueberries. Previous studies have shown that plant-derived extracts and phenolic compounds can contribute to the retention of fruit quality through antioxidant and antimicrobial effects. However, most conventional coating systems rely on casting or direct application approaches, whereas electrospun nanofibers provide a distinct high-surface-area architecture for incorporating and delivering plant-derived functional compounds [2,3,4,5].

Despite the growing interest in electrospun active packaging, the application of phenolic-rich *M. sylvestris* L. leaf extract in PVA-based electrospun nanofibers for blueberry preservation remains insufficiently investigated. Previous work has explored *M. sylvestris* L. constituents in other packaging-related applications, including the incorporation of its anthocyanins into chitosan–carrageenan electrospun structures for intelligent packaging [10]. However, the development of phenolic-rich *M. sylvestris* L. leaf-extract nanofibers and their combined characterization in terms of fiber morphology, phenolic composition, antioxidant functionality, antimicrobial activity, and practical performance during blueberry storage has received limited attention.

Accordingly, the present study aimed to develop phenolic-rich PVA-based electrospun nanofibers incorporating *M. sylvestris* L. leaf extract and to investigate their potential as active packaging materials for blueberry preservation. The developed nanofibers were characterized with respect to their morphology and functional properties, including antioxidant and antimicrobial activities. Their practical preservation performance was subsequently evaluated by monitoring the visual quality of blueberries during refrigerated storage. By integrating material characterization with a food-preservation application, this study seeks to provide evidence for the potential use of *M. sylvestris* L. phenolic compounds in electrospun active packaging systems for fresh fruit.

## 2. Results and Discussion

### 2.1. Fourier Transform Infrared Spectroscopy (FTIR)

**Figure 1:**
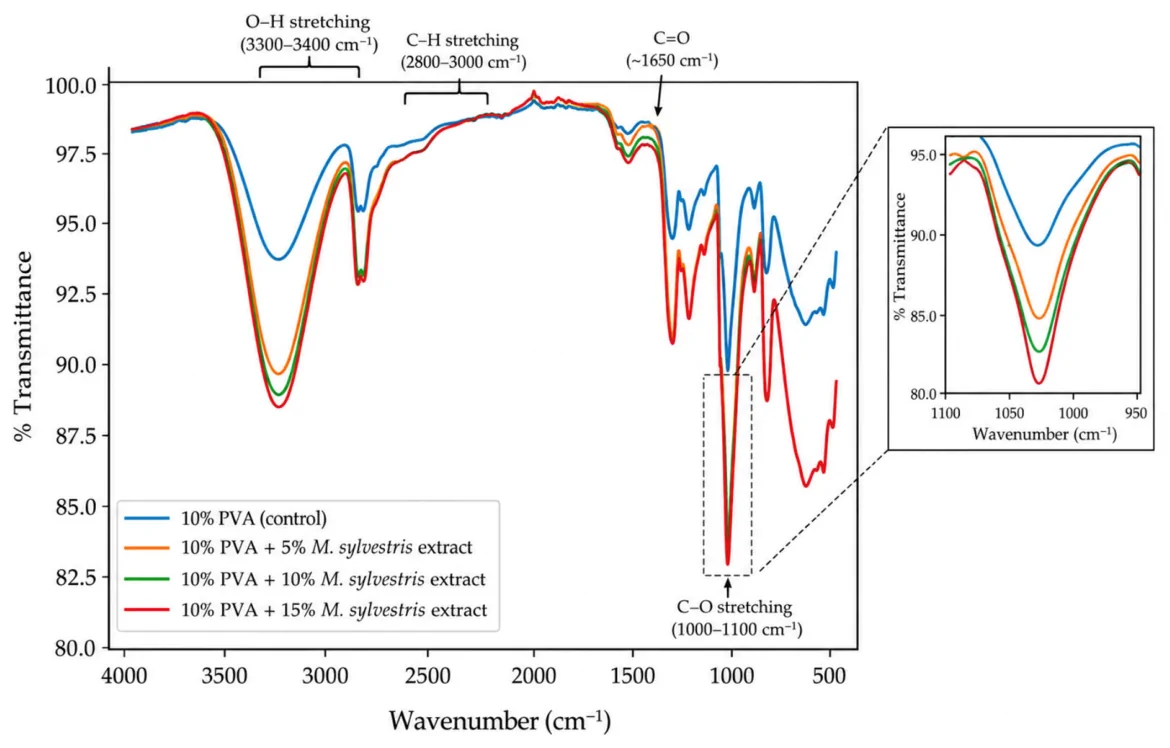
FTIR spectra of the electrospun nanofiber formulations: 10% PVA (control), 10% PVA + 5% *M. sylvestris* L. extract, 10% PVA + 10% *M. sylvestris* L. extract, and 10% PVA + 15% *M. sylvestris* L. extract, recorded in transmittance mode over the range of 4000–400 cm^−1^. The inset shows a magnified view of the 1000–1100 cm^−1^ region to facilitate comparison of the spectral differences among the formulations.

The FTIR spectra of the PVA control and *M. sylvestris* L. extract-loaded nanofibers are shown in Figure 1. All formulations exhibited a broad absorption feature in the approximately 3300–3400 cm^−1^ region, attributable primarily to O–H stretching vibrations. Compared with the PVA control, the extract-containing formulations showed a more pronounced feature in this region; however, the spectra of the 5%, 10%, and 15% extract-loaded formulations were largely superimposed. Therefore, the spectra do not support a clear concentration-dependent change in the O–H region.

More distinct differences among the formulations were observed within the fingerprint region, particularly around 1000–1100 cm^−1^, where the spectral profiles showed progressively greater differentiation with extract incorporation. This region includes contributions from C–O stretching vibrations associated with PVA and oxygen-containing functional groups of the plant-derived constituents. A magnified view of the 1000–1100 cm^−1^ region has therefore been included in Figure 1 to facilitate visualization of these differences. Quantitative comparisons of selected functional-group regions were performed by trapezoidal integration, with the resulting values normalized to the 2800–3000 cm^−1^ aliphatic C–H stretching region as a relative reference for intersample comparison.

Following the incorporation of *M. sylvestris* L. extract, the principal characteristic bands of PVA remained detectable in all nanofiber formulations, indicating that the basic chemical structure of the PVA matrix was maintained. Nevertheless, differences in the intensity and broadness of some absorption regions were observed as the extract concentration increased. In particular, the broad O–H stretching region at approximately 3300–3400 cm^−1^ became more pronounced in the extract-containing nanofibers. This change may be associated with the contribution of hydroxyl-containing constituents of the plant extract, particularly phenolic compounds, as well as changes in the hydrogen-bonding environment within the PVA matrix. Changes were also observed in the regions around 1600–1650 cm^−1^ and 1000–1100 cm^−1^ following extract incorporation. These spectral variations may reflect contributions from functional groups associated with the phytochemical constituents of *M. sylvestris* L., including aromatic and C–O-containing structures. The concentration-dependent changes in these regions are consistent with the increasing incorporation of extract-derived constituents into the nanofiber formulations. This interpretation is also supported by the HPLC and total phenolic content results, which demonstrated the presence of phenolic compounds in the plant extract and increasing phenolic content in the extract-containing nanofibers, respectively. Importantly, no distinct new absorption bands or major peak shifts were observed after incorporation of the extract. Therefore, the FTIR spectra do not provide evidence for the formation of new covalent bonds between PVA and the extract constituents. Rather, the observed changes are consistent with non-covalent intermolecular interactions, particularly hydrogen bonding, between the hydroxyl groups of PVA and hydroxyl-containing phytochemicals. Such interactions are plausible because both PVA and the phenolic constituents of the extract contain functional groups capable of participating in hydrogen bonding.

Overall, the FTIR results support the incorporation of *M. sylvestris* L.-derived constituents into the PVA nanofiber matrix while indicating that the characteristic chemical structure of PVA was largely preserved. The observed concentration-dependent spectral changes suggest modifications in the intermolecular hydrogen-bonding environment rather than the formation of new chemical bonds. These findings complement the HPLC and total phenolic content analyses and provide further evidence for the presence of plant-derived bioactive constituents within the electrospun nanofiber formulations.

Phenolic compounds present in plant extracts contain multiple hydroxyl groups that can interact with PVA chains through hydrogen bonding, thereby influencing solution viscosity, polymer chain entanglement, and jet stability during the electrospinning process. These interactions play a critical role in determining the morphology and diameter distribution of the resulting nanofibers. Previous studies have demonstrated that the incorporation of phenolic-rich plant extracts into electrospun polymeric nanofibers significantly affects fiber morphology and diameter owing to extract–polymer interactions. Appropriate extract concentrations generally promote the formation of uniform bead-free fibers, whereas excessive loading may adversely affect electrospinnability and lead to morphological defects [16,17,18,19,20].

### 2.2. Morphology of Electrospun Nanofibers

**Figure 2:**
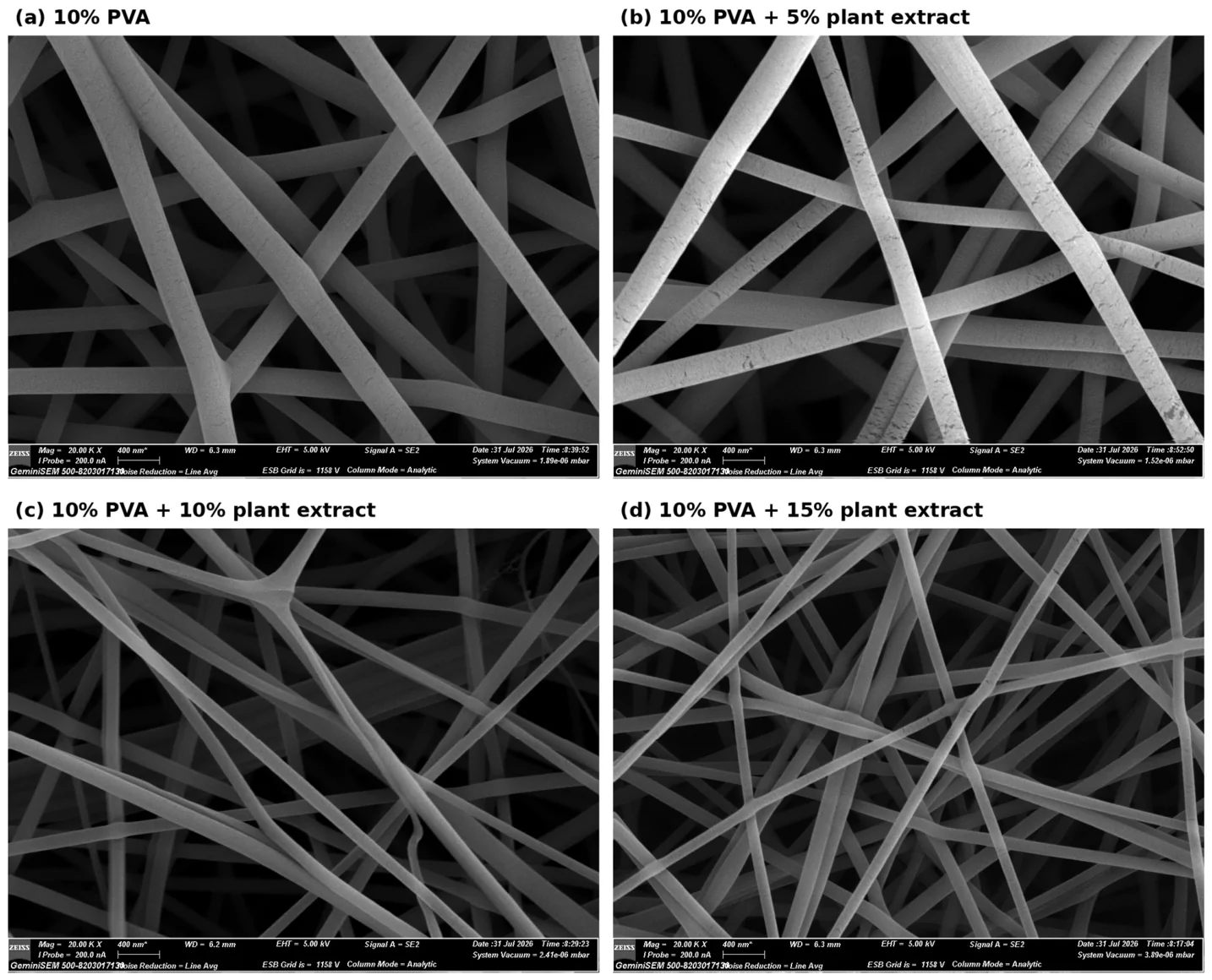
SEM images of electrospun nanofibers (**a**) 10% PVA (**b**) 10% PVA + 5% plant extract (**c**) 10% PVA + 10% plant extract (**d**) 10% PVA + 15% plant extract. All micrographs were obtained at 20,000× magnification and are presented with the same 400 nm scale bar *.

Scanning electron microscopy (SEM) was employed to investigate the morphology and fiber formation of electrospun PVA nanofibers containing different concentrations of *M. sylvestris* L. leaf extract (Figure 2). All samples exhibited smooth, randomly oriented, and bead-free fibers, indicating that the selected electrospinning parameters were appropriate for producing uniform nanofibrous mats. Quantitative analysis of the SEM images showed that the mean fiber diameter decreased progressively with increasing *M. sylvestris* L. extract concentration. The PVA-only nanofibers exhibited the largest mean fiber diameter (702 ± 76 nm), whereas incorporation of 5%, 10%, and 15% *M. sylvestris* L. extract resulted in mean fiber diameters of 565 ± 68 nm, 427 ± 52 nm, and 353 ± 38 nm, respectively (Table 1). This behavior may be associated with changes in the physicochemical properties of the electrospinning solution following the incorporation of the plant extract. *M. sylvestris* L. is rich in phenolic and other polar compounds containing hydroxyl groups, which can interact with the hydroxyl groups of PVA through hydrogen bonding. Such interactions may modify polymer–polymer interactions and the rheological behavior of the spinning solution, thereby influencing fiber formation during electrospinning.

Overall, the SEM observations confirmed the successful incorporation of *M. sylvestris* L. extract into the PVA nanofiber matrix without adversely affecting fiber formation, demonstrating the suitability of the developed electrospinning formulation for active food packaging applications.

### 2.3. Phenolic Profile of Plant Extract

The phenolic profile of plant extract was determined by HPLC, and the quantified phenolic compounds are presented in Table 2.

**Figure 3:**
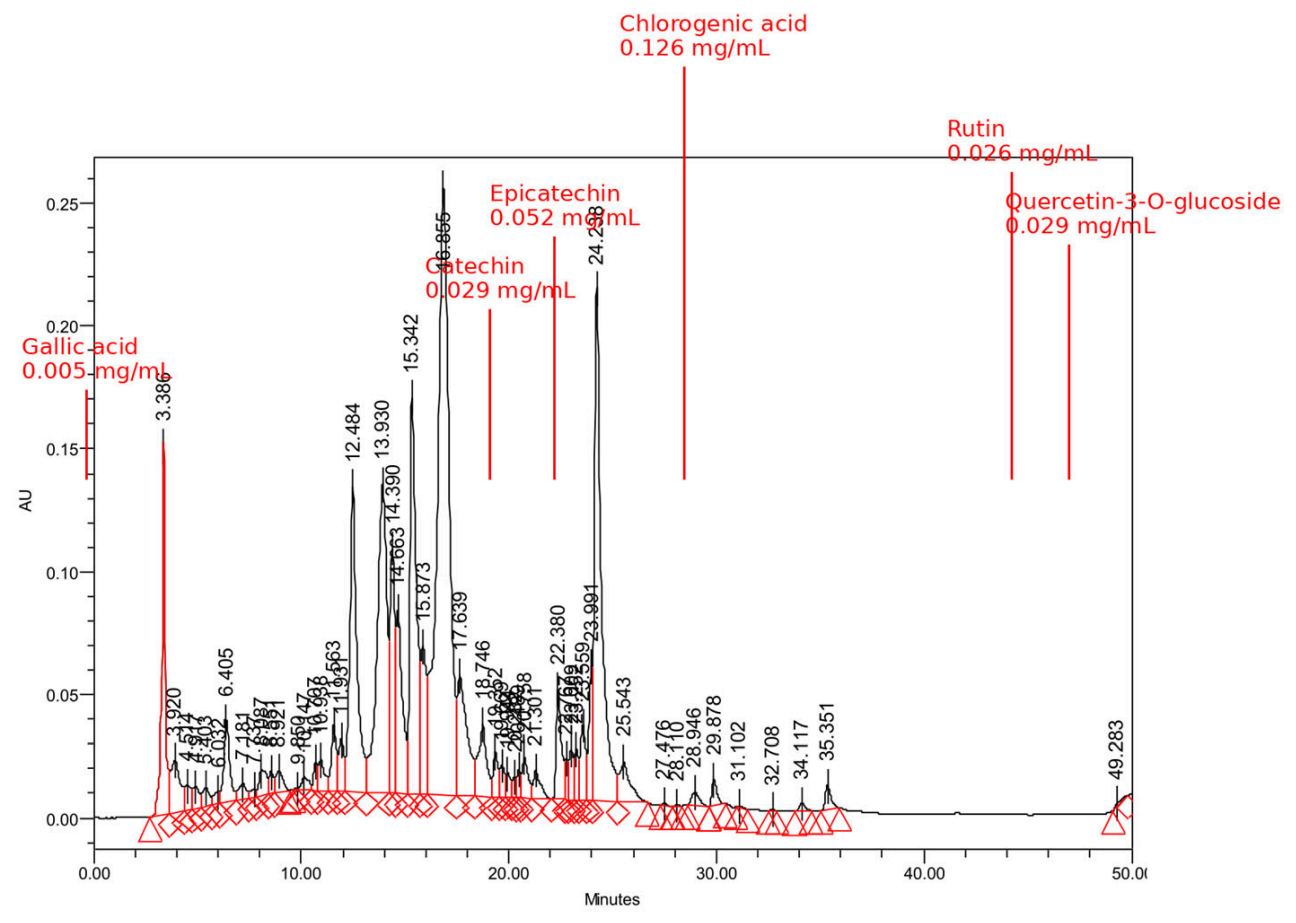
HPLC chromatogram of *M. sylvestris* L. leaf extract recorded at 280 nm.

The HPLC chromatogram of the *M. sylvestris* L. extract is presented in Figure 3. Chlorogenic acid was the major phenolic compound (0.126 mg/mL extract), followed by epicatechin (0.052 mg/mL), catechin and quercetin-3-O-glucoside (0.029 mg/mL each), rutin (0.026 mg/mL), and gallic acid (0.005 mg/mL), while protocatechuic acid was not detected. The predominance of chlorogenic acid, together with flavonoids such as catechin, epicatechin, rutin, and quercetin-3-O-glucoside, indicates that the extract is a rich source of antioxidant phytochemicals. These phenolic compounds are known for their free radical scavenging, antimicrobial, and metal-chelating properties, which likely contributed to the antioxidant and antimicrobial activities observed for the electrospun nanofibers. The HPLC results therefore confirm the suitability of *M. sylvestris* L. extract as a natural bioactive ingredient for active food packaging applications.

### 2.4. Total Phenolic Content

The total phenolic content (TPC) of the electrospun nanofibers increased significantly with increasing concentrations of *M. sylvestris* L. extract (Table 3). However, the values should not be interpreted as encapsulation efficiencies because the phenolic content of the spinning solutions prior to electrospinning was not quantified on an equivalent basis.

The total phenolic content of the electrospun nanofibers increased significantly with increasing *M. sylvestris* L. extract concentration (*p* < 0.05) (Table 3). One-way ANOVA followed by Tukey’s HSD test showed significant differences among all formulations. No detectable phenolic content was observed in the PVA control, whereas the nanofibers containing 5%, 10%, and 15% extract exhibited TPC values of 6.49 ± 0.09, 11.66 ± 0.33, and 17.76 ± 0.29 mg GAE/g, respectively. Thus, increasing the extract concentration from 5% to 15% resulted in an approximately 2.7-fold increase in the measured TPC of the resulting nanofibers.

The concentration-dependent increase demonstrates that increasing the amount of *M. sylvestris* L. extract in the electrospinning formulation resulted in a correspondingly greater phenolic content in the obtained nanofibers. This trend is also relevant to the antioxidant and antibacterial results, since the formulations with higher measured TPC generally exhibited greater biological activity. In particular, the 15% formulation, which had the highest TPC, also showed the strongest antioxidant and antibacterial performance among the investigated nanofiber formulations.

However, the TPC values represent the phenolic compounds detected in the resulting nanofibers and should not be interpreted as encapsulation efficiency or direct evidence that phenolic compounds were fully retained during electrospinning. Because the phenolic content of the spinning solutions was not quantified on an equivalent basis before electrospinning, potential phenolic losses during processing could not be determined. Nevertheless, the results clearly demonstrate a significant extract-concentration-dependent increase in the phenolic content of the electrospun nanofibers.

Several previous studies have reported that electrospinning is an effective technique for encapsulating and protecting phenolic compounds against degradation. Unlike conventional thermal processing methods, electrospinning is generally performed at ambient temperature and atmospheric pressure, thereby minimizing the thermal and chemical degradation of sensitive bioactive compounds. Consequently, electrospun nanofibers have been widely proposed as promising delivery systems for polyphenols and other thermolabile phytochemicals [21,22,23,24].

### 2.5. Antioxidant Activity

#### 2.5.1. DPPH Radical Scavenging Activity

The incorporation of *M. sylvestris* L. extract markedly increased the DPPH radical-scavenging activity of the electrospun nanofibers. The PVA-only nanofibers exhibited a low DPPH scavenging activity of 4.8 ± 0.5%, whereas the values increased to 39.6 ± 1.3%, 63.8 ± 1.7%, and 82.9 ± 2.0% for the nanofibers containing 5%, 10%, and 15% *M. sylvestris* L. extract, respectively (Table 4). Thus, the 15% extract-loaded formulation exhibited the highest DPPH radical-scavenging activity (82.9 ± 2.0%), demonstrating a concentration-dependent enhancement in antioxidant capacity with increasing extract incorporation.

#### 2.5.2. ABTS Radical Scavenging Activity

A similar concentration-dependent trend was observed for the ABTS radical-scavenging assay. The PVA-only nanofibers showed relatively low ABTS scavenging activity (6.2 ± 0.6%), whereas incorporation of 5%, 10%, and 15% *M. sylvestris* L. extract increased the activity to 46.8 ± 1.5%, 70.5 ± 1.9%, and 89.7 ± 2.3%, respectively. The 15% formulation exhibited the highest ABTS scavenging activity (89.7 ± 2.3%), confirming that increasing the amount of *M. sylvestris* L. extract enhanced the radical-scavenging capacity of the electrospun nanofibers.

#### 2.5.3. Ferric Reducing Antioxidant Power (FRAP)

The FRAP results further supported the concentration-dependent enhancement of antioxidant activity following incorporation of *M. sylvestris* L. extract. The PVA-only nanofibers exhibited a FRAP value of 42.5 ± 3.8 μmol Fe^2+^/g, which increased substantially to 286.7 ± 11.4, 487.9 ± 15.8, and 694.2 ± 18.5 μmol Fe^2+^/g for the 5%, 10%, and 15% extract-loaded nanofibers, respectively. The highest reducing capacity was therefore observed for the 15% formulation (694.2 ± 18.5 μmol Fe^2+^/g). This progressive increase is consistent with the DPPH and ABTS results and supports the contribution of the incorporated phenolic constituents to the antioxidant functionality of the nanofiber matrix. The FRAP results further demonstrated that phenolic compounds retained their reducing capacity following electrospinning. Previous studies have reported similar findings for electrospun nanofibers incorporating plant extracts rich in flavonoids and phenolic acids, demonstrating that electrospinning is an effective strategy for encapsulating plant-derived bioactive compounds while preserving their antioxidant properties and ensuring their homogeneous distribution within polymeric matrices. The incorporation of phenolic-rich extracts has been shown to improve the functional properties of electrospun nanofibers without compromising fiber formation when appropriate extract concentrations are employed. These bioactive nanofibrous systems have attracted considerable attention for food packaging and biomedical applications owing to their enhanced antioxidant, antimicrobial, and controlled-release characteristics [25,26,27,28,29,30].

The marked improvement in antioxidant activity can be attributed to the high content of phenolic acids, flavonoids, anthocyanins, and tannins naturally present in *M. sylvestris* L. These phytochemicals readily donate hydrogen atoms or electrons to neutralize free radicals and reduce Fe^3+^ to Fe^2+^, thereby improving both radical scavenging activity and reducing power. The progressive enhancement observed with increasing extract concentration indicates successful incorporation of the bioactive compounds into the electrospun PVA matrix.

Electrospinning is also known to preserve the biological activity of plant-derived antioxidants because fiber formation occurs at ambient temperature and solvent evaporation is extremely rapid, minimizing thermal degradation of phenolic compounds. In addition, the high specific surface area and porous morphology of electrospun nanofibers facilitate rapid interaction between encapsulated antioxidants and reactive oxygen species. The absence of a decrease in antioxidant activity after electrospinning suggests that the PVA matrix effectively immobilized the extract while maintaining the functionality of its phenolic constituents [31,32,33,34,35,36].

### 2.6. Antimicrobial Activity

The antibacterial activity of *M. sylvestris* L. plant extract against *E. coli* and *S. aureus* are presented in Table 5.

The antibacterial activity of *M. sylvestris* L. extract against *E. coli* and *S. aureus* is presented in Table 5. The extract inhibited both microorganisms, producing inhibition zones of 2.15 ± 0.07 cm against *E. coli* and 2.28 ± 0.05 cm against *S. aureus*. Although the difference was relatively small, the larger inhibition zone against *S. aureus* indicated slightly greater susceptibility of the Gram-positive bacterium to the extract. This trend was supported by the MIC results, for which the free extract required 16 μg/mL against *E. coli* but only 8 μg/mL against *S. aureus*. Thus, both antibacterial assays consistently indicated greater activity against *S. aureus*. The difference may be partly related to differences in cell-envelope structure, as the outer membrane of Gram-negative bacteria such as *E. coli* can restrict the penetration of some antimicrobial phytochemicals. The antibacterial activity of *M. sylvestris* L. has been attributed to its high content of phenolic compounds, flavonoids, and tannins, which can disrupt bacterial cell membranes, interfere with cellular metabolism, and inhibit microbial growth. Similar antibacterial effects of *M. sylvestris* L. extracts against both Gram-positive and Gram-negative bacteria have been reported previously, supporting the potential application of this plant as a natural antimicrobial agent for food preservation and active packaging applications [37,38,39,40].

The antibacterial activity of the electrospun nanofibers was dependent on the concentration of *M. sylvestris* L. extract incorporated into the PVA matrix (Table 6).

The 5% extract-loaded nanofibers exhibited MIC values of 64 μg/mL against *E. coli* and 32 μg/mL against *S. aureus*. Increasing the extract concentration to 10% reduced these values to 32 and 16 μg/mL, respectively, corresponding to a two-fold improvement in antibacterial activity against both microorganisms. The 15% extract-loaded nanofibers exhibited the strongest antibacterial activity among the electrospun formulations, with MIC values comparable to those obtained for the free plant extract. The progressive decrease in MIC values with increasing extract concentration demonstrates that higher loading of phenolic compounds, flavonoids, and tannins enhances the antibacterial effectiveness of the nanofibers. Although expression of the nanofiber MICs on an extract-equivalent basis facilitates comparison with the free extract, these results should not be interpreted as demonstrating identical antimicrobial behavior of the two systems. Incorporation into the PVA matrix may influence the accessibility and release of the bioactive compounds. Therefore, the comparable MIC values observed for the 15% formulation and the free extract indicate that substantial antibacterial activity was retained following incorporation into the electrospun nanofiber matrix. The incorporation of bioactive compounds into the electrospun PVA matrix may modulate their availability and potentially enhance their stability by limiting direct exposure to environmental factors; however, sustained-release behavior and protection against phytochemical degradation cannot be confirmed without dedicated release kinetics and stability studies. Consequently, the 15% *M. sylvestris* L. loaded electrospun nanofibers showed the greatest antibacterial potential and appear to be the most promising formulation for active food-packaging applications, where prolonged antimicrobial activity is desirable.

### 2.7. Blueberry Preservation Study

#### Visual Quality and Preservation During Refrigerated Storage

The visual quality of blueberries was evaluated at the predefined assessment points of Days 0, 7, and 14 during refrigerated storage. At Day 0, the blueberries exhibited excellent initial visual quality, with intact surfaces, characteristic coloration, and no visible signs of shriveling or fungal deterioration. During storage, clear differences became evident between the untreated control and nanofiber-packaged groups. By Day 7, the control blueberries showed marked deterioration in visual appearance, including increased shriveling and visible fungal development, whereas the nanofiber-packaged blueberries maintained a comparatively intact external appearance (Table 7).

**Figure 4:**
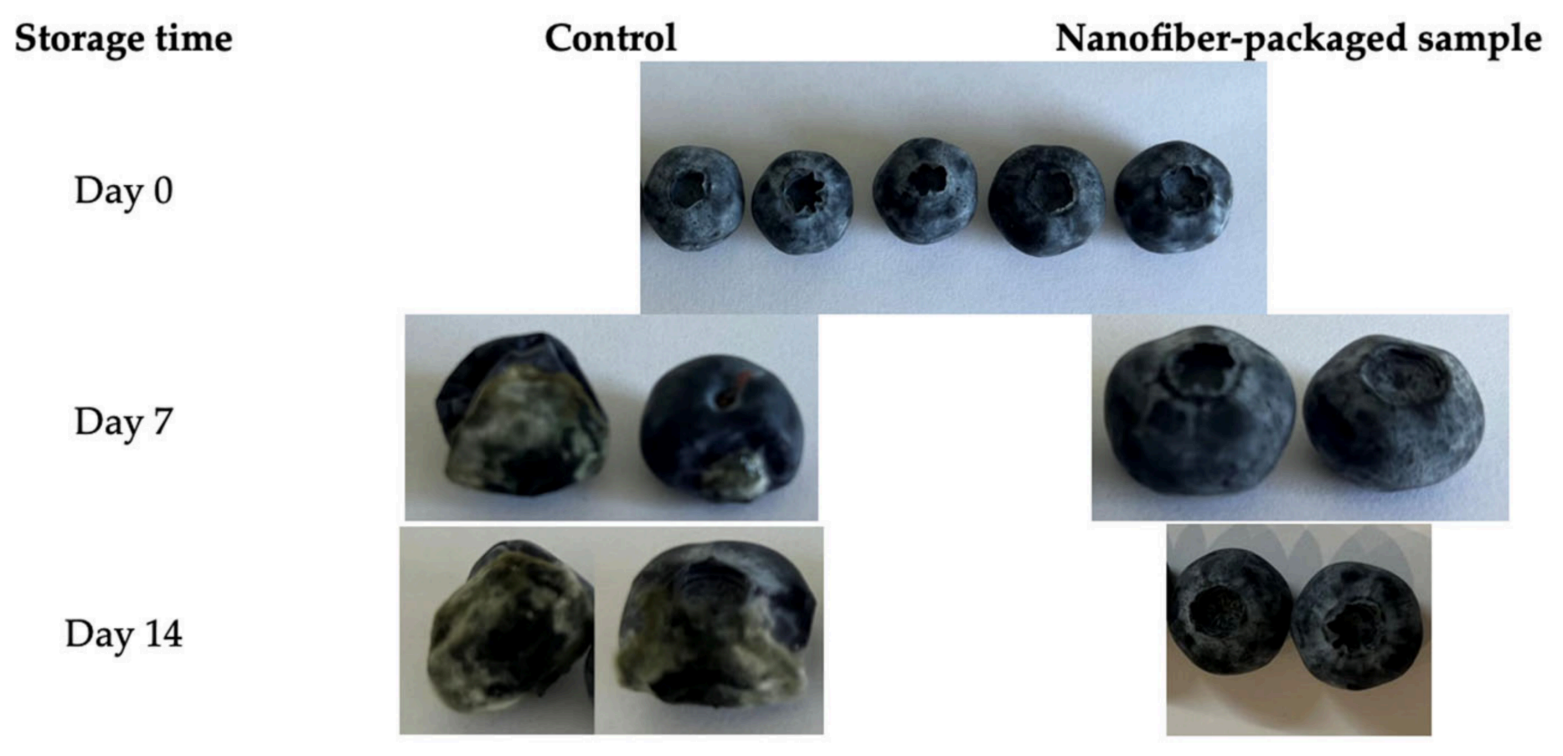
Representative visual appearance of blueberries during refrigerated storage.

Representative photographs of the blueberries during refrigerated storage are presented in Figure 4. Day 7 was the first scheduled evaluation point at which clear visible deterioration was observed in the untreated control blueberries, including visible fungal development and loss of normal external appearance. In contrast, the nanofiber-packaged blueberries retained a comparatively intact appearance at the same evaluation point. By Day 14, deterioration of the control fruit had become more pronounced, whereas the nanofiber-packaged samples retained substantially better visual quality.

These photographic observations were consistent with the quantitative visual-quality scores presented in Table 7. At Day 7, the control group had a mean visual-quality score of 2.4 ± 0.55, which was below the predefined commercial acceptability threshold of 3, whereas the nanofiber-packaged group maintained a score of 4.8 ± 0.45. By Day 14, the visual-quality score of the control group had further decreased to 1.6 ± 0.55, while the nanofiber-packaged group retained a score of 4.2 ± 0.45 and remained within the commercially acceptable range. Thus, the differences between the two groups became increasingly apparent during refrigerated storage.

Overall, the electrospun nanofiber packaging treatment delayed visible quality deterioration and contributed to maintaining the visual acceptability of the blueberries during the investigated 14-day refrigerated-storage period. The nanofiber-packaged fruit exhibited less apparent shriveling and visible fungal deterioration than the untreated control, consistent with the higher visual-quality scores observed at Days 7 and 14. However, because shelf life was not determined using a comprehensive set of physicochemical, microbiological, and sensory endpoints, these findings should be interpreted as evidence of delayed visible quality deterioration and improved maintenance of visual quality under the investigated conditions, rather than as a demonstrated extension of marketable shelf life.

## 3. Materials and Methods

### 3.1. Materials

**Figure 5:**
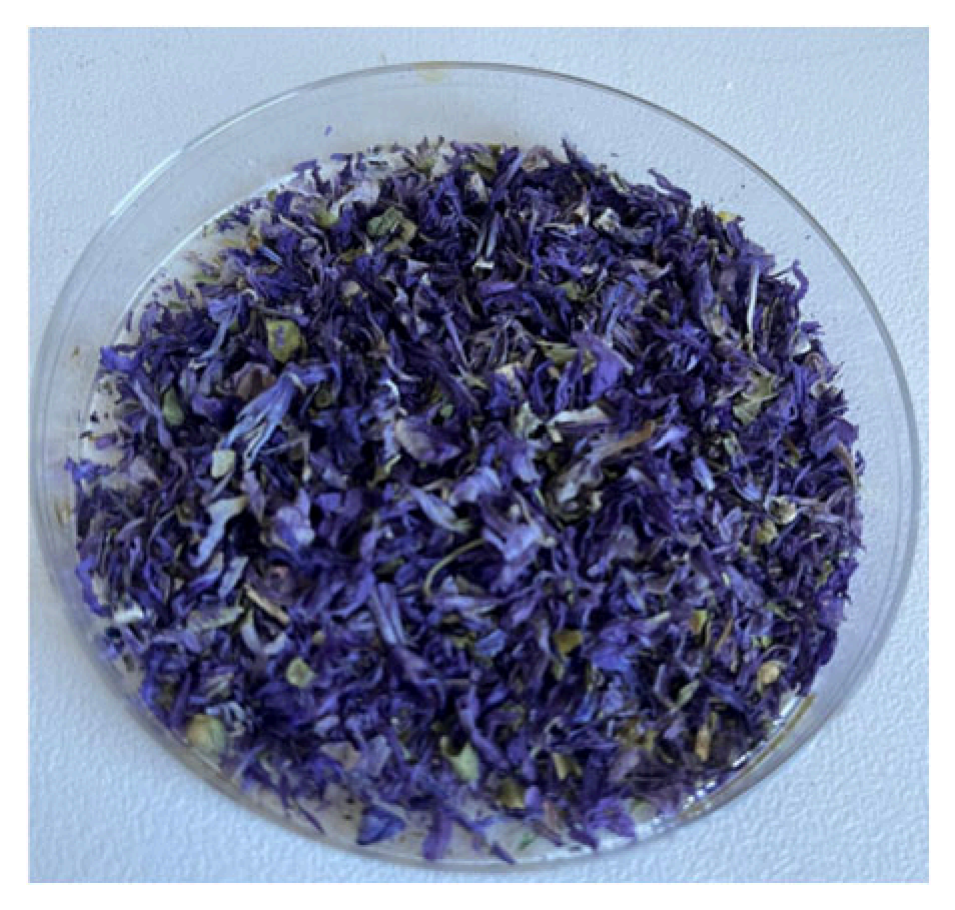
*M. sylvestris* L. plant.

Polyvinyl alcohol (PVA; Mw 85,000–124,000 g mol^−1^; ≥99% hydrolyzed) (CAS No. 9002-89-5) was purchased from Sigma-Aldrich (St. Louis, MO, USA). Polyvinyl alcohol (PVA) (CAS No. 9002-89-5) was purchased from Sigma-Aldrich (St. Louis, MO, USA). *M. sylvestris* L. (Figure 5) were bought from Istanbul, Türkiye local markets. Ethanol (analytical grade) (CAS No. 64-17-5), methanol (CAS No. 67-56-1), Folin–Ciocalteu reagent, gallic acid (CAS No. 149-91-7), 2,2-diphenyl-1-picrylhydrazyl (DPPH) (CAS No. 1898-66-4), potassium persulfate (CAS 7727-21-1), 2,2′-azinobis-(3-ethylbenzothiazoline-6-sulfonic acid) (ABTS) (CAS No. 30931-67-0), ferric chloride (CAS No. 7705-08-0), potassium ferricyanide (CAS No. 13746-66-2), trichloroacetic acid (CAS No. 76-03-9), ferric reducing antioxidant power (FRAP) reagent, and all other analytical-grade chemicals were obtained from Sigma-Aldrich (St. Louis, MO, USA) unless otherwise stated.

Fresh blueberries (*Vaccinium corymbosum* L.) at commercial maturity, free from mechanical damage and visible fungal infection, were purchased from a local commercial producer in Istanbul, Türkiye in June 2026 and immediately transported to the laboratory for the preservation experiments.

### 3.2. Methods

#### 3.2.1. Preparation of *M. sylvestris* L. Leaf Extract

Prior to extraction, *M. sylvestris* L. plant material was rinsed thoroughly with distilled water to eliminate surface contaminants. The cleaned samples were then allowed to dry in the shade at ambient temperature until no further change in weight was observed. After drying, the plant material was pulverized using a laboratory mill to obtain a homogeneous fine powder.

The powdered material was combined with 70% (*v*/*v*) ethanol at a solid-to-solvent ratio of 1:10 (*w*/*v*). Extraction was carried out by ultrasound-assisted extraction for 60 min, with the temperature maintained at approximately 4 °C. To limit the temperature increase associated with sonication, the ultrasonic bath was cooled using an ice bath, and ice was replaced when necessary. The extraction temperature was monitored throughout the procedure to ensure that low-temperature conditions were maintained. Following sonication, the plant–solvent mixture was passed through Whatman No. 1 filter paper to separate the liquid extract from the remaining plant material. Ethanol was subsequently removed from the filtrate under reduced pressure using a rotary evaporator operated at 40 °C. The resulting concentrated extract was transferred to suitable containers and maintained at −20 °C until subsequent analyses and preparation of the electrospinning formulations.

These extraction conditions were chosen to promote efficient recovery of phenolic constituents while limiting their exposure to elevated temperatures, thereby reducing the potential degradation of thermolabile bioactive compounds. Similar hydroethanolic extraction approaches have previously been applied to Malva species and other phenolic-rich plant materials [6,8,41].

#### 3.2.2. Determination of Phenolic Profile by HPLC

The phenolic composition of the plant extract was determined by high-performance liquid chromatography (HPLC) following the procedure described by Capanoglu et al. [42], with all measurements conducted in triplicate. Prior to chromatographic analysis, the extract was passed through a 0.45 µm membrane filter and subsequently introduced into the HPLC system. The chromatographic system was equipped with a Waters 600 controller and a Waters 996 photodiode array (PDA) detector. Separation of the phenolic constituents was achieved using a C18 reversed-phase column.

The mobile phase consisted of two solvents: 0.01% trifluoroacetic acid (TFA) in water (solvent A) and 0.01% TFA in acetonitrile (solvent B). Chromatographic separation was performed at a flow rate of 1.0 mL/min using a linear gradient in which the proportion of acetonitrile was increased from 5% to 35% over a total run time of 50 min. The eluted phenolic compounds were monitored with the PDA detector at 280, 312, and 360 nm. Quantification was performed based on the chromatographic responses obtained at the appropriate detection wavelength for each phenolic compound [42].

#### 3.2.3. Preparation of Electrospinning Solutions

An aqueous PVA solution (10%, *w*/*v*) was obtained by dispersing the polymer powder in distilled water and heating the mixture at 90 °C for 2 h under constant magnetic agitation. Stirring was continued until the PVA was completely dissolved and a clear, uniform solution was formed. The prepared polymer solution was then cooled to room temperature before the addition of *M. sylvestris* L. extract.

Three extract-containing formulations were produced by adding the plant extract at levels of 5%, 10%, and 15% (*w*/*w*), calculated relative to the dry weight of PVA. Following extract addition, each formulation was continuously stirred at ambient temperature for 2 h to achieve uniform distribution of the extract throughout the polymeric phase. Prior to electrospinning, the solutions were subjected to ultrasonic treatment for 15 min to eliminate entrapped air and obtain bubble-free spinning formulations [1,5].

In parallel, an extract-free 10% (*w*/*v*) PVA formulation was processed under the same conditions and used as the control. Thus, four electrospinning formulations were prepared: PVA without extract and PVA supplemented with 5%, 10%, or 15% *M. sylvestris* L. extract.

The selected extract concentrations enabled evaluation of the effect of increasing bioactive incorporation on the structural characteristics and antioxidant functionality of the resulting nanofibers, as well as their performance during refrigerated storage of blueberries.

#### 3.2.4. Electrospinning Process

Electrospinning was performed using a syringe-pump/high-voltage setup equipped with a grounded rotating drum collector. The prepared spinning solutions were loaded into a 10 mL syringe fitted with a 21-gauge stainless-steel needle. The applied voltage, solution flow rate, and needle-to-collector distance were maintained at 18 kV, 0.5 mL h^−1^, and 15 cm, respectively. The rotating drum collector was operated at 1000 rpm and covered with aluminum foil for nanofiber collection. Electrospinning was conducted at an ambient temperature of 25 °C and a relative humidity of 45%. Following electrospinning, the collected nanofiber mats were maintained overnight at room temperature and subsequently stored under dry conditions until further analysis [1,43].

#### 3.2.5. Scanning Electron Microscopy (SEM)

The morphology and structural characteristics of the electrospun fiber surfaces were evaluated by scanning electron microscopy (SEM) using a Zeiss Gemini 500 instrument (Zeiss, Oberkochen, Germany). For SEM examination, small sections of the nanofibrous mats were fixed onto aluminum specimen stubs with double-sided conductive carbon tape. The mounted samples were subsequently coated with a thin gold layer by sputtering under vacuum to minimize surface charging and improve image quality.

SEM images were recorded at several magnifications using accelerating voltages within the range of 5–15 kV. In addition to qualitative examination of fiber morphology, the micrographs were subjected to quantitative analysis to determine fiber size and its distribution. Fiber diameters were measured using ImageJ software (Version 1.54t; National Institutes of Health, Bethesda, MD, USA). For each formulation, a minimum of 100 fibers were randomly chosen from different and spatially separated areas of the SEM micrographs to minimize selection bias and provide representative measurements of the nanofibrous structure.

The fiber-diameter data are reported as mean ± standard deviation. Representative SEM images used for the quantitative diameter measurements are presented in the Supplementary Material (Figure S1).

#### 3.2.6. Fourier Transform Infrared Spectroscopy (FTIR)

Fourier transform infrared (FTIR) spectroscopy was employed to characterize the functional groups present in the electrospun nanofibrous mats and to assess chemical changes associated with the incorporation of *M. sylvestris* L. extract into the PVA matrix. Spectral measurements were obtained at ambient temperature over the range of 4000–400 cm^−1^. The spectra of the extract-containing nanofibers were compared with those of the PVA control by evaluating the position and profile of the major absorption bands. Particular attention was given to variations in characteristic bands that could reflect molecular interactions between PVA and the phytochemical constituents of the plant extract. Spectral evaluation was conducted with reference to previously reported FTIR characteristics of PVA-based electrospun systems [44,45].

#### 3.2.7. Preparation of Nanofiber Extracts for Phenolic and Antioxidant Analyses

Prior to total phenolic content (TPC) and antioxidant analyses, 25 mg of each electrospun nanofiber mat was accurately weighed and transferred into a sealed vial containing 20 mL of ethanol/water (70:30, *v*/*v*). The samples were maintained under continuous stirring at 50 °C for 24 h to facilitate exhaustive extraction of phenolic and antioxidant compounds entrapped within the nanofiber matrix. Following extraction, the mixtures were centrifuged at 5000 rpm for 10 min to separate residual polymeric material and suspended debris. The resulting clear supernatants were carefully collected and used for the TPC, DPPH, ABTS, and FRAP assays. The PVA-only nanofiber mat was subjected to the same extraction procedure and served as the polymer-matrix control. All samples were prepared under identical extraction conditions.

#### 3.2.8. Determination of Total Phenolic Content (TPC)

The total phenolic content (TPC) of the nanofiber extracts prepared as described above was determined using the modified Folin–Ciocalteu assay according to the procedure reported previously [46]. For the analysis, 0.5 mL of the appropriately diluted sample was combined with 2.5 mL of Folin–Ciocalteu reagent previously diluted tenfold with distilled water. The reaction mixture was allowed to stand for 5 min, after which 2.0 mL of sodium carbonate solution (7.5%, *w*/*v*) was introduced. The samples were then maintained for 30 min at ambient temperature in the absence of light to allow color development.

Following incubation, the optical absorbance of each reaction mixture was recorded at 765 nm using a UV–Visible spectrophotometer (Thermo Fisher Scientific, Waltham, MA, USA). Quantification was carried out using a gallic acid calibration curve, and TPC values were reported as milligrams of gallic acid equivalents per gram of dry sample (mg GAE/g). Each sample was analyzed in triplicate, and the corresponding mean values were used for subsequent evaluation.

#### 3.2.9. DPPH Radical Scavenging Activity

The nanofiber extracts prepared as described above were used for the DPPH radical-scavenging assay. The radical-scavenging capacity of the samples was assessed using the DPPH (2,2-diphenyl-1-picrylhydrazyl) assay, based on the method originally described by Brand-Williams et al. [31] with appropriate modifications. A 0.1 mM DPPH working solution was prepared freshly in methanol immediately before analysis. For the reaction, 0.5 mL of each sample extract was combined with 2.5 mL of the freshly prepared DPPH solution. The mixtures were thoroughly mixed and maintained at room temperature for 30 min under dark conditions to prevent light-induced degradation of the radical. After incubation, the decrease in DPPH absorbance was recorded at 517 nm using a UV–Visible spectrophotometer.

The radical scavenging activity was calculated according to Equation (1). All analyses were conducted in triplicate.

DPPH scavenging activity (%) = (A_0_ − A_s_)/A_0_ × 100 (1)

where A_0_ and A_s_ represent the absorbance of the control and the sample, respectively.

#### 3.2.10. ABTS Radical Scavenging Activity

The nanofiber extracts prepared as described above were used for the ABTS radical-scavenging assay. The antioxidant capacity of the samples was further evaluated by the ABTS radical cation decolorization assay, adapted from the procedure reported by Re et al. [32]. To generate the ABTS radical cation (ABTS•+), an aqueous ABTS solution (7 mM) was combined with potassium persulfate (2.45 mM). The reaction mixture was maintained at ambient temperature for 16 h in the absence of light to allow complete radical formation.

Immediately before use, the resulting ABTS•+ solution was diluted with ethanol until an absorbance of 0.70 ± 0.02 at 734 nm was reached. For each measurement, 0.1 mL of the sample was introduced into 3.9 mL of the adjusted ABTS•+ working solution and mixed thoroughly. Following a 6 min reaction period, the absorbance was recorded at 734 nm using a UV–Visible spectrophotometer. The ABTS radical scavenging activity was calculated as the percentage inhibition of the ABTS•+ using the following equation (Equation (2)). All measurements were performed in triplicate.

ABTS radical scavenging activity (%) = [(A_0_ − A_s_)/A_0_] × 100 (2)

where A_0_ represents the absorbance of the control and A_s_ represents the absorbance of the sample.

#### 3.2.11. Ferric Reducing Antioxidant Power (FRAP)

The nanofiber extracts prepared as described above were used for the FRAP assay. The reducing capacity of the samples was evaluated using the ferric reducing antioxidant power (FRAP) assay based on the procedure established by Benzie and Strain [33]. The working FRAP reagent was prepared immediately before use by combining acetate buffer (300 mM, pH 3.6), 2,4,6-tripyridyl-s-triazine (TPTZ; 10 mM in 40 mM HCl), and FeCl_3_·6H_2_O solution (20 mM) at a volumetric ratio of 10:1:1.

For the assay, 100 μL of each sample was added to 3.0 mL of freshly prepared FRAP reagent. The reaction mixtures were maintained at 37 °C for 30 min to allow development of the colored Fe^2+^–TPTZ complex. Following incubation, absorbance was measured at 593 nm using a UV–Visible spectrophotometer. Quantification was performed against a calibration curve prepared with ferrous sulfate, and the reducing capacity was reported as μmol Fe^2+^ equivalents per gram of dry sample (μmol Fe^2+^ equivalents/g). All determinations were carried out in triplicate.

#### 3.2.12. Antimicrobial Activity

The antibacterial potential of *M. sylvestris* L. extract was initially assessed by the agar disc diffusion technique using the Gram-positive bacterium *Staphylococcus aureus* and the Gram-negative bacterium *Escherichia coli* as test microorganisms. Freshly prepared bacterial suspensions were standardized to 0.5 McFarland, corresponding to approximately 10^8^ CFU/mL, and evenly inoculated onto the surface of Mueller–Hinton agar (MHA) plates. Sterile filter-paper discs (6 mm in diameter) were then positioned on the inoculated agar, and 10 µL of the plant extract was loaded onto each disc. Two extract-loaded discs were used per Petri dish. Following incubation at 37 °C for 24 h, antibacterial activity was evaluated by measuring the diameter of the clear zones formed around the discs using a digital caliper. The assay was conducted in triplicate [47,48].

The minimum inhibitory concentrations (MICs) of the plant extract and electrospun nanofiber samples were assessed by a broth microdilution procedure in sterile 96-well microplates, with reference to the Clinical and Laboratory Standards Institute (CLSI) recommendations and the ISO 20776-1 broth microdilution method [49,50]. For the nanofiber formulations, MIC were expressed on an extract-equivalent mass basis, representing the mass of *M. sylvestris* extract incorporated within the tested nanofiber material per unit volume of broth (μg extract equivalent/mL). For the free plant extract, concentrations were expressed as μg extract/mL. Two-fold serial dilutions of the test samples were prepared in Mueller–Hinton broth (MHB), covering a concentration range of 256–0.098 μL/mL. Each experimental well contained 180 µL of the corresponding sample dilution and 20 µL of bacterial suspension, resulting in a total volume of 200 µL.

Optical density at 600 nm (OD_600_) was recorded using a microplate reader (BioTek Technologies, Winooski, VT, USA) both before and after incubation at 35 °C for 24 h. Wells containing bacterial inoculum in MHB without test material were included as growth controls, whereas wells containing the corresponding test sample in MHB without bacterial inoculation were included to account for sample-related background. The MIC was assigned as the lowest tested concentration at which no visible bacterial growth was detected following the incubation period [49,50].

The growth-inhibitory response observed in the microdilution assay was additionally verified using resazurin as a metabolic viability indicator. Following the MIC incubation period, 10 µL of resazurin solution (1 mg/mL) was introduced into each well, and the microplates were incubated for an additional 3 h at 35 °C. The assay is based on the reduction in resazurin by metabolically active bacterial cells, which produces a visible transition from blue to pink. Accordingly, wells that remained blue were interpreted as showing inhibition of bacterial metabolic activity, whereas development of a pink coloration indicated the presence of viable, metabolically active bacterial cells [51,52].

#### 3.2.13. Blueberry Preservation Study

Fresh blueberries (*Vaccinium corymbosum* L.) were screened immediately following purchase to obtain a relatively homogeneous batch for the storage experiment. Fruits of comparable size, coloration, and maturity were selected, whereas berries showing visible physical damage or signs of fungal deterioration were excluded. The selected fruits were randomly allocated to the control and nanofiber-packaged groups.

For refrigerated storage, untreated blueberries were transferred to sterile polyethylene terephthalate (PET) containers and maintained as the control group. For the packaged group, the electrospun nanofiber mats were positioned within the packages to cover the fruit surface and function as active packaging material. All experimental packages were maintained at 4 °C and 90% relative humidity (RH) for 14 days. Visual-quality assessments were performed at three predefined time points: Day 0, Day 7, and Day 14. At each assessment point, changes in surface color, shriveling, visible fungal development, and overall visual quality were evaluated [53].

Visual quality was independently assessed by five trained evaluators using a five-point categorical scale, where 5 = excellent, 4 = good, 3 = acceptable, 2 = poor, and 1 = unacceptable. Each evaluator assigned one integer score (1–5) to each treatment at each evaluation time point. Results are expressed as the mean ± standard deviation of the five individual evaluator scores (*n* = 5). A mean score below 3 was considered indicative of commercially unacceptable visual quality [54].

#### 3.2.14. Statistical Analysis

All experiments were performed in triplicate, and the results were expressed as mean ± standard deviation. Statistical analysis was conducted using SPSS software (version 20.0, SPSS Inc., Chicago, IL, USA). Data were analyzed using one-way analysis of variance (ANOVA). Differences among treatment means were evaluated using Tukey’s multiple comparison test at a significance level of *p* < 0.05.

## 4. Conclusions

The present study demonstrated the successful development of phenolic-rich electrospun PVA nanofiber mats incorporating *M. sylvestris* L. extract as a bioactive packaging material. Incorporation of the plant extract improved the functional characteristics of the electrospun matrix, resulting in concentration-dependent increases in phenolic content, antioxidant activity, and antibacterial activity. Among the investigated formulations, nanofibers containing 15% *M. sylvestris* L. extract exhibited the strongest functional performance. During refrigerated blueberry storage, the nanofiber packaging treatment helped maintain visual quality and delayed visible deterioration compared with the untreated control. These findings support the potential of *M. sylvestris* L.-loaded electrospun PVA nanofiber mats as active packaging materials for fresh produce. Nevertheless, further studies addressing food-contact safety, migration behavior, release kinetics, physicochemical quality parameters, and microbiological shelf-life criteria are required before their suitability for practical food-packaging applications and their ability to extend product shelf life can be established.

## Figures and Tables

**Table 1:** The average fiber diameter according to the concentrations of plant extract.

Plant Extract Concentration %	Fiber Diameter (nm) ^1^
0	702 ± 76 ^a^
5	565 ± 68 ^b^
10	427 ± 52 ^c^
15	353 ± 38 ^d^

^1^ Values are expressed as mean ± standard deviation (*n* = 100). Different superscript letters indicate significant differences, *p* < 0.05.

**Table 2:** The phenolic profile of plant extract.

Compounds	Sample (mg/mL Extract)
Gallic acid	0.005 ± 0.0006
Protocatechuic acid	ND
Epicatechin	0.052 ± 0.0015
Catechin	0.029 ± 0.0011
Chlorogenic acid	0.126 ± 0.0022
Rutin	0.026 ± 0.0016
Quercetin-3-O-glucoside	0.029 ± 0.0012

Values are expressed as mean ± standard deviation (SD) of three replicate measurements (*n* = 3). ND: not detected.

**Table 3:** TPC of electrospun nanofibers.

Sample	TPC (mg GAE/g Nanofiber) ^1^
10% PVA	ND
10% PVA + 5% *M. sylvestris* L. extract	6.49 ± 0.09 ^c^
10% PVA + 10% *M. sylvestris* L. extract	11.66 ± 0.33 ^b^
10% PVA + 15% *M. sylvestris* L. extract	17.76 ± 0.29 ^a^

^1^ Values are expressed as mean ± standard deviation (*n* = 3). ND: not detected under the analytical conditions used. Different superscript letters indicate significant differences among the quantitatively determined extract-containing formulations (*p* < 0.05; one-way ANOVA followed by Tukey’s multiple-comparison test). The PVA control was not included in the mean-separation analysis because no quantifiable TPC value was obtained.

**Table 4:** Antioxidant activity of electrospun PVA nanofibers containing different concentrations of *M. sylvestris* L. extract, determined by DPPH, ABTS, and FRAP assays.

Sample	DPPH (%)	ABTS (%)	FRAP (µmol Fe^2+^/g)
10% PVA nanofiber	4.8 ± 0.5 ^a^	6.2 ± 0.6 ^a^	42.5 ± 3.8 ^a^
10% PVA + 5% *M. sylvestris* L. extract	39.6 ± 1.3 ^b^	46.8 ± 1.5 ^b^	286.7 ± 11.4 ^b^
10% PVA + 10% *M. sylvestris* L. extract	63.8 ± 1.7 ^c^	70.5 ± 1.9 ^c^	487.9 ± 15.8 ^c^
10% PVA + 15% *M. sylvestris* L. extract	82.9 ± 2.0 ^d^	89.7 ± 2.3 ^d^	694.2 ± 18.5 ^d^

Values are expressed as mean ± standard deviation (*n* = 3). Different superscript letters within the same column indicate statistically significant differences among formulations according to Tukey’s HSD test at *p* < 0.05.

**Table 5:** Antibacterial Activity and Inhibitory Zone Diameters (cm) of Plant Extract.

Plant Extract	*E. coli*	*S. aureus*
*M. sylvestris* L.	2.15 ± 0.07	2.28 ± 0.05
	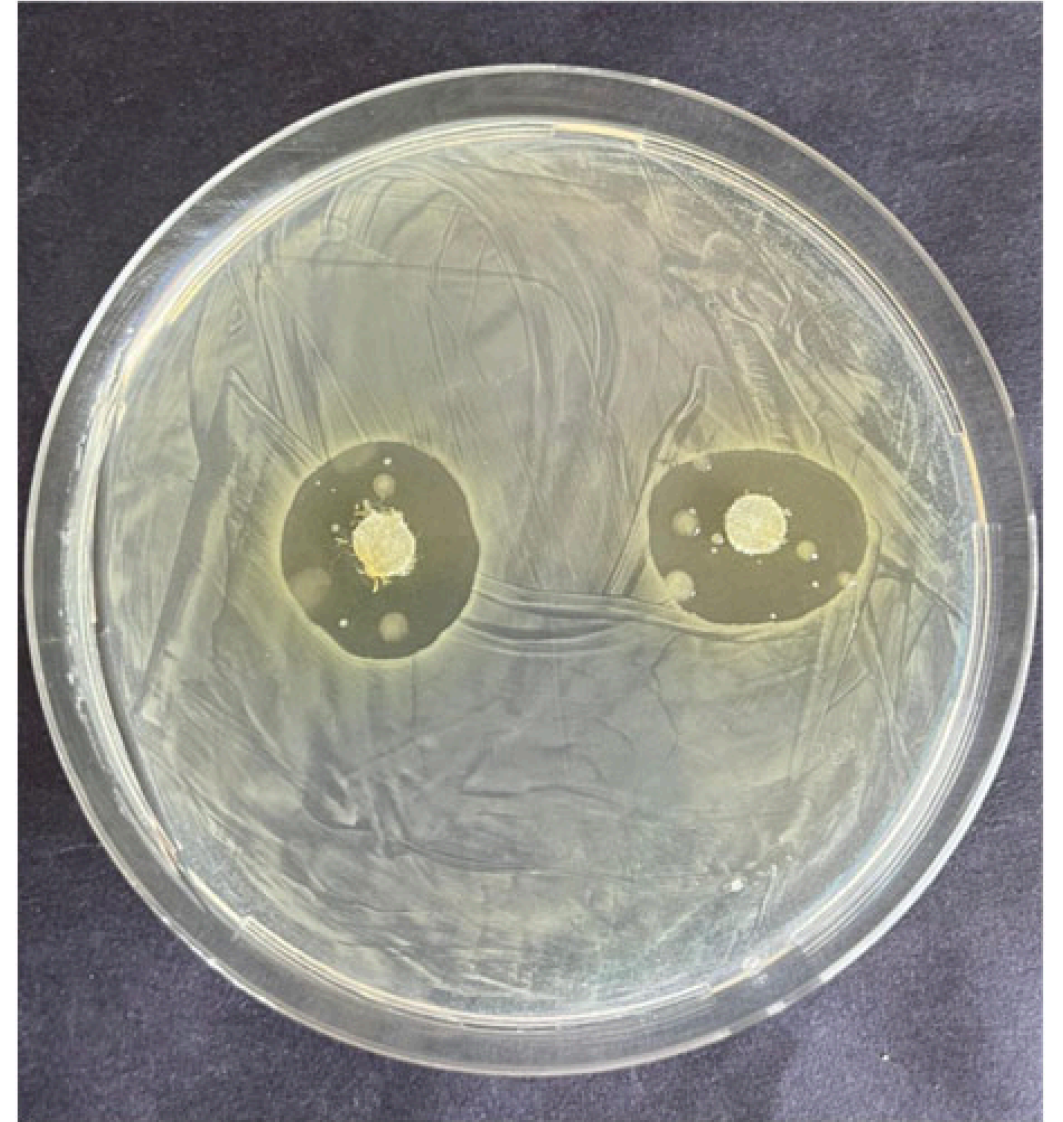	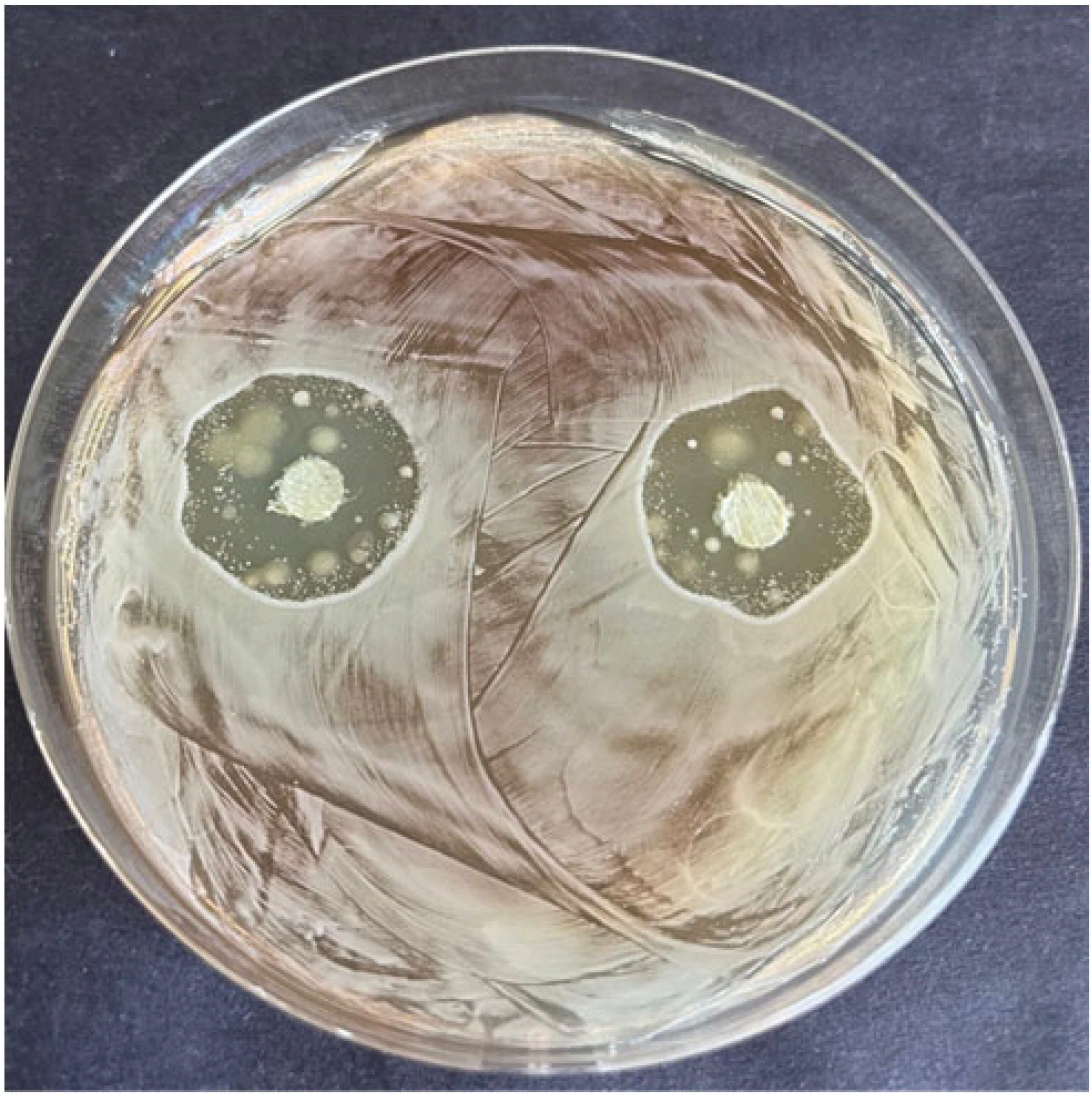

Values are expressed as mean ± standard deviation (*n* = 3).

**Table 6:** Minimum inhibitory concentrations of free *M. sylvestris* L. extract and extract-loaded PVA electrospun nanofiber formulations against *S. aureus* and *E. coli*.

Samples	*E. coli*	*S. aureus*
Plant extract	16	8
5% electrospun nanofiber	64	32
10% electrospun nanofiber	32	16
15% electrospun nanofiber	16	8

Note: For the free *M. sylvestris* extract, concentrations represent μg extract/mL. For extract-loaded nanofiber formulations, concentrations are expressed on an extract-equivalent basis (μg extract equivalent/mL), calculated from the nominal amount of *M. sylvestris* extract incorporated into each nanofiber formulation.

**Table 7:** Quantitative visual-quality scores of blueberries.

Storage Time	Control ^1^	Nanofiber-Packaged Sample ^1^	Commercial Interpretation
Day 0	5.0 ± 0.00	5.0 ± 0.00	Excellent
Day 7	2.4 ± 0.55	4.8 ± 0.45	Control: unacceptable; treated: excellent
Day 14	1.6 ± 0.55	4.2 ± 0.45	Control: unacceptable; treated: good

^1^ Values are expressed as mean ± standard deviation (*n* = 5). Visual quality was evaluated using a five-point scale (5 = excellent, 4 = good, 3 = acceptable, 2 = poor, and 1 = unacceptable); mean scores below 3 were considered commercially unacceptable.

## Data Availability

The original contributions presented in this study are included in the article/Supplementary Material. Further inquiries can be directed to the corresponding author.

## Supplementary Materials

The following supporting information can be downloaded at: https://www.mdpi.com/article/10.3390/molecules31183283/s1, Figure S1: SEM micrographs of electrospun nanofibers at different magnifications (1000×, 5000×, 10,000×, 20,000×, and 40,000) (a) 10% PVA (b) 10% PVA + 5% plant extract (c) 10% PVA + 10% plant extract (d) 10% PVA + 15% plant extract.

## Abbreviations

The following abbreviations are used in this manuscript:

ABTS	2,2′-Azinobis-(3-ethylbenzothiazoline-6-sulfonic acid)
DPPH	2,2-Diphenyl-1-picrylhydrazyl
FRAP	Ferric Reducing Antioxidant Power
GAE	Gallic acid equivalents
HPLC	High Performance Liquid Chromatography
PET	Polyethylene terephthalate
PVA	Polyvinyl alcohol
RH	Relative humidity
TFA	Trifluoroacetic acid
TPC	Total phenolic content
TPTZ	2,4,6-tripyridyl-s-triazine
