# Supplementary material for: Phenolic-Rich Electrospun Bioactive Nanofibers from Mallow (Malva sylvestris L.) as Active Packaging Materials
Source: Molecules. 2026 Sep 16;31(18):3283. doi: 10.3390/molecules31183283 (PMC13609245; doi:10.3390/molecules31183283)
Supplement: Supplementary file 1 [file molecules-31-03283-s001.zip › molecules-4509693-supplementary.pdf]

Supplementary Material

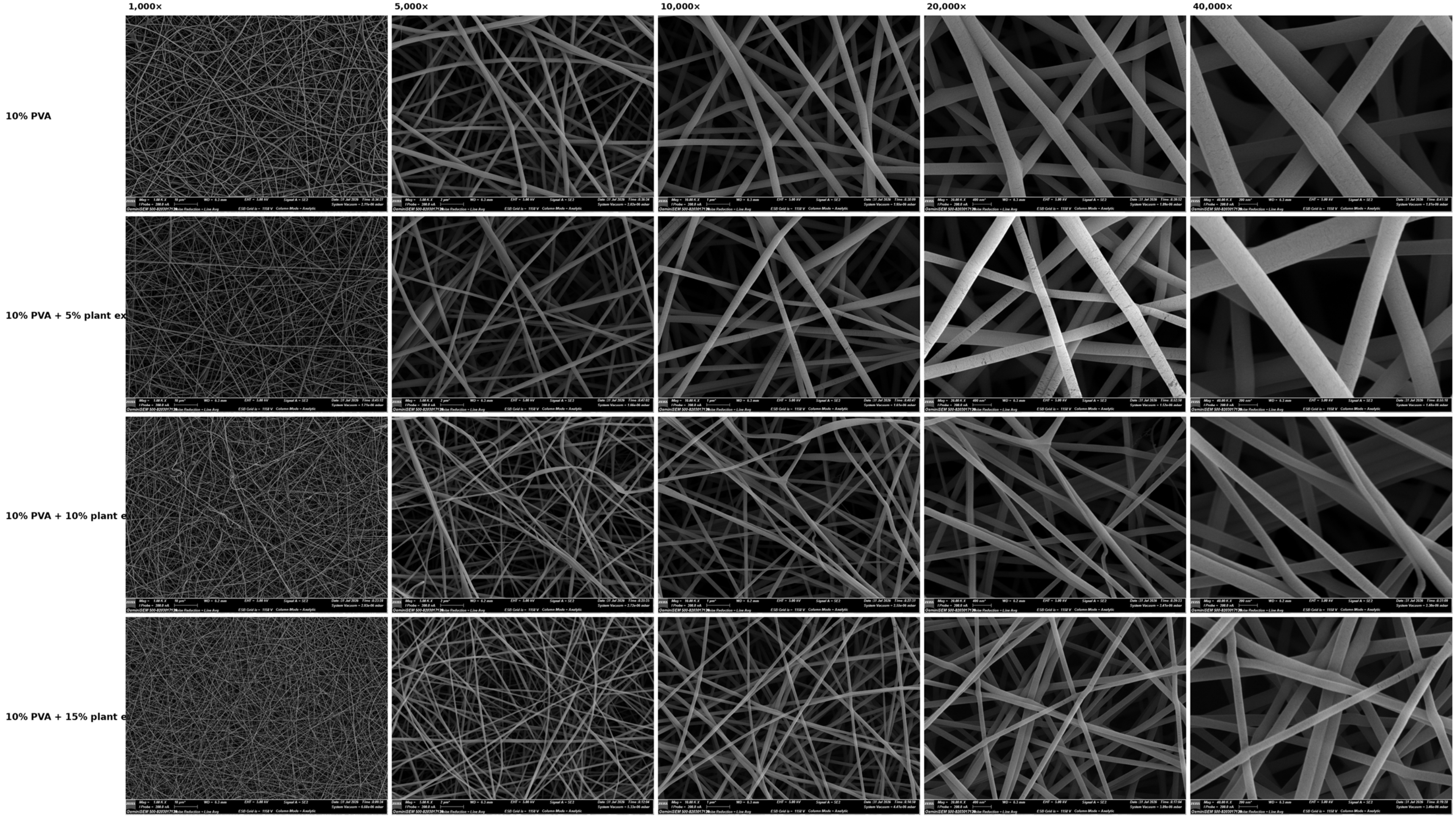

Figure S1. SEM micrographs of electrospun nanofibers at different magnifications (1000×, 5000×, 10,000×, 20,000×, and 40,000) (a) 10% PVA (b) 10% PVA + 5% plant extract (c) 10% PVA + 10% plant extract (d) 10% PVA + 15% plant extract.
